# Polyomaviruses After Allogeneic Hematopoietic Stem Cell Transplantation

**DOI:** 10.3390/v17030403

**Published:** 2025-03-12

**Authors:** Maria Alejandra Mendoza, Hannah Imlay

**Affiliations:** Division of Infectious Diseases, Department of Internal Medicine, University of Utah, Salt Lake City, UT 84112, USA; alejandra.mendoza@hsc.utah.edu

**Keywords:** JC virus, BK virus, polyomavirus, hematopoietic stem cell transplant, progressive multifocal leukoencephalopathy, hemorrhagic cystitis

## Abstract

Polyomaviruses (PyVs) are non-enveloped double-stranded DNA viruses that can cause significant morbidity in allogeneic hematopoietic stem cell transplant (allo-HSCT) recipients, particularly BK polyomavirus (BKPyV) and JC polyomavirus (JCPyV). BKPyV is primarily associated with hemorrhagic cystitis (HC), while JCPyV causes progressive multifocal leukoencephalopathy (PML). The pathogenesis of these diseases involves viral reactivation under immunosuppressive conditions, leading to replication in tissues such as the kidney, bladder, and central nervous system. BKPyV-HC presents as hematuria and urinary symptoms, graded by severity. PML, though rare after allo-HSCT, manifests as neurological deficits due to JCPyV replication in glial cells. Diagnosis relies on nucleic acid amplification testing for DNAuria or DNAemia as well as clinical criteria. Management primarily involves supportive care, as no antiviral treatments have proven consistently effective for either virus and need further research. This review highlights the virology, clinical presentations, and management challenges of PyV-associated diseases post-allo-HSCT, emphasizing the need for improved diagnostic tools and therapeutic approaches to mitigate morbidity and mortality in this vulnerable population.

## 1. Introduction

Polyomaviruses (PyVs) are ubiquitous and can infect human and other animal hosts. The best described PyVs are BK polyomavirus (BKPyV) and JC polyomavirus (JCPyV), which were named after patients with BKPyV-associated ureteric obstruction after kidney transplantation and progressive multifocal leukoencephalopathy (PML) after treatment of lymphoma, respectively [1,2]. After allogeneic hematopoietic stem cell transplant (allo-HSCT), PyVs can replicate asymptomatically or cause morbid disease. BKPyV can cause hemorrhagic cystitis (BKPyV-HC) in allo-HSCT recipients, and although JCPyV has been commonly associated with PML in other patient populations (classically among those with HIV/AIDS or patients receiving specific immunomodulators), PML has also been reported after HSCT.

In this review, we will primarily focus on BKPyV-associated diseases as BKPyV-HC is uniquely associated with allogeneic HSCT, and to a lesser extent JCPyV-associated disease; we will also briefly discuss reports of other PyVs causing disease following allo-HSCT. Due to a paucity of data, some information is extrapolated from other immunocompromised hosts such as kidney transplant recipients. We will refer to detection of PyVs in the blood and urine as “DNAemia” and “DNAuria”, respectively.

Human polyomaviruses (HPyVs) have been linked with transformation to malignancy [3], including the association between BKPyV with bladder and urothelial carcinoma [4,5,6,7], Merkel cell PyV (MCPyV) as a cause of Merkel cell carcinoma [8], and JCPyV with a several solid tumors [9]. For the purposes of this review, we will not discuss the association between HPyVs and malignancy, but this topic has been reviewed elsewhere [3,10,11].

### Virology of Polyomaviruses

PyVs are non-enveloped double stranded DNA (dsDNA) viruses within the family Polyomaviridae that infect humans and animals [12]. BKPyV and JCPyV share 70–75% identity across their genomes and are closely related to SV-40. The genome of all three viruses contains two transcriptional units separated by a non-coding control region (NCCR) that contains the origin of replication and promoters/enhancers for early and late gene expression. One transcriptional unit encodes the early genes: large T antigen, small t antigen, and T antigen splice variants (depending on which PyV). The other transcriptional unit encodes the late genes: three structural proteins (VP1, VP2, and VP3), and agnoprotein, and two micro RNAs that downregulate large T antigen [13]. Rearrangements in NCCR sequences occur in vivo and are thought to be responsible for or associated with progression to some PyV-diseases (e.g., PML and BKPyV-Associated Nephropathy [BKPyVAN] after kidney transplant) but findings are mixed in others (e.g., BKPyV-HC) [13,14,15,16]. Specific mechanisms of cell entry and viral replication within the cell have been reviewed extensively elsewhere [13,17].

In renal or other tissues, a cross-reacting antibody to SV40-LTag can identify either BKPyV or JCPyV and virus-specific nucleic acid amplification testing (NAAT) is required to distinguish between them [18]. In HSCT recipients, the diagnosis of BKPyV and JCPyV replication relies on detection of replication using NAAT assays obtained from cerebrospinal fluid (CSF), serum, or urine. Importantly, since there is inter-assay variability (due to differences in amplicon size, sample type, target, detection of unencapsidated DNA, etc.) and challenges with the WHO international standard, absolute levels of DNAuria (viruria) or DNAemia (viremia) cannot be directly compared across assays, which has limited the ability to establish diagnostic criteria that are based on absolute DNA load thresholds [16,19,20,21]. Methods to increase precision and compare results between assays are a major focus of ongoing work, including the ability to distinguish replicating virus from DNA shedding [16,18,22,23].

## 2. BKPyV Syndromes After Allo-HSCT

BKPyV-associated clinical syndromes are more common than PML following allo-HSCT. Among BKPyV-associated syndromes, hemorrhagic cystitis (BKPyV-HC) is best characterized, however, analogous to JCPyV, BKPyV DNAuria and DNAemia are common. 

### 2.1. Clinical Presentation of BKPyV Syndromes and Incidence After HSCT

a. Asymptomatic DNAuria

BKPyV DNAuria after HSCT is common [24]. In studies of HSCT recipients who undergo regular monitoring, BKPyV DNAuria increases from <10% before HSCT to 50–100% after transplant, typically identified between 1–8 weeks post-HSCT [25,26,27,28]. Progression to BKPyV-HC occurs in a minority of patients with DNAuria but is associated with high urinary BKPyV loads [24,26,27,29].

b. Asymptomatic DNAemia

In studies that prospectively monitored patients after allo-HSCT, BKPyV DNAemia was reported in 50–80% of pediatric patients [30,31] and 20–60% of adults, with a median onset of 30–40 days post-HSCT [32,33,34,35]. Reactivation and detection of dsDNA viruses, including BKPyV, were independently associated with increased risk for both early and late mortality after accounting for immune reconstitution, acute graft versus host disease (GVHD) severity, and steroid use [36]; the majority of virus reactivations occurred without clinical symptoms.

c. BKPyV-associated hemorrhagic cystitis:

The incidence of BKPyV-HC in patients after allo-HSCT patients varies across studies; incidence after allogeneic HSCT in adults is 16% (range, 7–54%) [25,26,27,32,35] and 18% (range, 7–25%) in children [37,38,39]. The variation in observed incidence may be due to differences in study populations, conditioning regimens, and diagnostic criteria followed by each study.

BKPyV-HC is generally characterized by hematuria, symptoms of UTI, and detection of BKPyV in urine. Other potential etiologies of cystitis should be ruled out, which can make diagnosis difficult in the presence of co-pathogens, which are common [40,41]. Hematuria is graded according to the Bedi et al. classification [26]:

- Grade 1: Microscopic hematuria
- Grade 2: Macroscopic hematuria
- Grade 3: Macroscopic hematuria with clots
- Grade 4: Macroscopic hematuria requiring instrumentation.

Stricter consensus diagnostic criteria were developed by ECIL that do not include Bedi grade 1 (microscopic) hematuria in the case definition:

1. Clinical symptoms/signs of cystitis, such as dysuria and lower abdominal pain;
2. Hematuria of grade 2 or higher;
3. Demonstration of BKPyV DNAuria, with viral loads exceeding 7 log10 copies/mL [37].

Irritative bladder symptoms most commonly include dysuria, frequency, or pelvic discomfort [35,37,40]. BKPyV-HC typically develops between 2 and 8 weeks post-hematopoietic cell transplantation, with a range from 1 week to 6 months [42]. The time to symptom resolution varies among studies. One retrospective analysis reported that the median time for macroscopic hematuria resolution was 17 days (range: 10–30 days), while the resolution of all symptoms, including cystitis, occurred at a median of 24 days (range: 15–44 days). In multivariable models, a high plasma viral load (≥10,000 copies/mL) and cytopenias at the onset of BKPyV-HC were significantly associated with prolonged macroscopic hematuria and cystitis symptoms [40]. Severe BKPyV-HC is characterized by hematuria and blood clots that can obstruct bladder or ureteral flow, causing obstructive kidney injury or requiring surgical intervention; the incidence of severe BKPyV-HC varies across studies, between 32–59% for HC grade 3 or higher [43,44]. The diagnosis of BKPyV-HC is associated with longer hospitalizations, more transfusions, and higher healthcare cost [45]. Importantly, some data indicate that the duration and severity of HC symptoms have no significant impact on progression-free survival [46], but results are mixed [35,47].

BKPyV loads in urine are significantly higher than in blood among patients with BKPyV-HC and not all cases of BKPyV-HC are accompanied by BKPyV DNAemia [40]. Relative to patients with asymptomatic DNAuria or DNAemia, patients with BKPyV-HC of any grade of hematuria have significantly higher BKPyV loads in urine or blood [48,49,50,51,52]; limited data suggest that BKPyV plasma loads are similar between different grades of hematuria [40]. BKPyV plasma loads of >10,000 copies/mL have been associated with higher BKPyV-HC severity and longer duration [40,45,48,53,54,55].

Since BKPyV replication can be detected a median of 8 days before the onset of BKPyV-HC [56], prospective monitoring has been proposed as a strategy to identify high-risk patients. If a future effective treatment is identified, monitoring could identify a high-risk group of patients who could benefit from pre-emptive intervention. Cesaro et al. conducted a prospective study of 107 pediatric HSCT recipients and demonstrated that plasma BKPyV-DNAemia monitoring outperformed urine BKPyV load in predicting BKPyV-HC. A plasma BKPyV DNA load of 10^3^ copies/mL exhibited 100% sensitivity, 86% specificity, a negative predictive value (NPV) of 100%, and a positive predictive value (PPV) of 39% for subsequent HC. In contrast, a urine BKPyV-DNA load > 10⁷ copies/mL showed 86% sensitivity, 60% specificity, an NPV of 98%, and a PPV of 14% for HC46. Similar findings have been reported in other pediatric studies [46,47,48,53]. Small studies in adults have also suggested that plasma monitoring of BKPyV-DNA may identify patients at risk of HC46,47,49. In one study, the presence of BKPyV-DNA in serum on day 21 post-HSCT (>0.75 × 10^3^ BKPyV copies/mL) was a statistically significant risk factor for HC and survival outcomes50. Another study demonstrated that plasma BKPyV PCR titters on days 0, 30, and 60 post-transplant were sensitive tools for predicting clinically significant HC51. A recent retrospective study demonstrated that high BKPyV viremia is associated with an early decline in renal function and worse survival [47].

Current guidelines from the European Conference on Infections in Leukemia (ECIL) do not recommend routine screening for BKPyV virus in the urine or blood in asymptomatic patients. BKPyV DNAuria is common in this population and even though the presence and degree of BKPyV DNAemia is linked with BKPyV-HC, detection of DNAemia is not sensitive for BKPyV-HC [37]. Furthermore, the lack of effective prevention or management strategies limits the actionability of routine surveillance.

d. Non-hemorrhagic cystitis syndromes associated with BKPyV

Cases of BKPyV non-hemorrhagic cystitis have been reported in non-allo-HSCT recipients [57,58] as a diagnosis of exclusion when no other urinary pathogens were identified. BKPyV cystitis without macroscopic hematuria, either with microscopic (grade 1) hematuria or without documented hematuria at all, are included in observational studies of BKPyV disease after allo-HSCT. For example, in a prospective study of 99 HSCT patients diagnosed with BKPyV disease, only 38% reported hematuria, while cystitis symptoms such as increased urinary frequency and dysuria were observed in 88% and 63% of cases, respectively [35]. Similarly, another study on BKPyV-associated cystitis identified dysuria as the most common symptom in 88.5%, followed by hematuria in 79% [43]. Microscopic (grade 1) hematuria has been reported in 5.7% to 30.2% of cases across various studies [40,45,59].

Along the same lines, a subset of retrospective study of 128 allo-HSCT recipients who ultimately developed BKPyV-HC at grade 2 or above found no statistically significant difference in viral load at time points of cystitis symptoms alone, cystitis with macroscopic hematuria, and cystitis with hematuria and clots [40]. In many patients, cystitis symptoms preceded hematuria [40,50]. However, the overall incidence of BKPyV-associated cystitis without micro or macroscopic hematuria following allo-HSCT remains unknown.

In immunocompetent patients with interstitial cystitis, the potential contribution of PyV has been proposed based on higher PyV detection compared to matched controls, however PyV detection is inconsistent in biopsy specimens and this association requires confirmation in larger studies [60,61,62,63,64].

e. BKPyV-associated nephropathy

BKPyVAN is the major complication of BKPyV replication after kidney transplant recipients (KTR) and is best characterized in this setting. In KTRs, BKPyV DNAemia is thought to be a result of BKPyV replication and cell lysis within the allograft [65]. Definitive diagnosis relies on allograft biopsy with demonstration of SV-40 staining, but high-level BKPyV DNAemia is clinically used as a tool for presumptive diagnosis [18,66,67]. BKPyVAN after KTR is a major cause of allograft dysfunction and loss [67].

Unlike after KTR, renal dysfunction among patients with BKPyV-HC is commonly caused by obstructive renal injury [24,48]. However, BKPyVAN after allo-HSCT has been reported [48,68,69,70], and renal dysfunction has been observed among HSCT recipients with BKPyV DNAemia even without known BKPyV-HC-related obstruction [47,48,71,72,73]. Several studies have demonstrated long-term renal dysfunction associated with BKPyV DNAemia > 10,000 copies/mL, a threshold that is associated with BKPyVAN after KT [18,48,66,67,72] Since renal biopsy is rarely feasible or safe in the post-HSCT period, the true incidence of BKPyVAN after HSCT, or whether or not it occurs concomitantly with BKPyV-HC, is unknown.

f. Cases of BKPyV replication linked to clinical disease

Other potential cases of BKPyV replication have occurred in patients with encephalitis, reported in allo-HSCT recipients with high BKPyV loads in CSF [74,75,76,77,78,79], and pneumonia diagnosed based on demonstration of viral inclusion bodies and SV-40 staining from lung tissue [80]. Some of these reported cases have occurred in patients with other concurrent manifestations of BKPyV such as HC or PyVAN. In vitro studies have demonstrated the potential for BKPyV to infect brain endothelial cells, ependymal cells, and astrocytes; however, the incidence of these syndromes, or whether BKPyV replication is a cause or a bystander in all of these cases is unknown [81,82,83,84].

The presence of BKPyV-HC has been statistically significant risk factor associated with complement-associated thrombotic microangiopathy, with a hazard ratio (HR) of 2.55 demonstrated in adults [85].

### 2.2. Pathogenesis of BKPyV Syndromes After HSCT

Analogous to JCPyV, primary infection with BKPyV occurs early in life and persists asymptomatically within the renourinary tract, including renal tubular epithelial cells, renal glomerular cells, and potentially bladder microvascular endothelial cells [86,87]. BKPyV replication in urine or plasma occurs intermittently in immunocompetent patients but increases following HSCT-associated immunosuppression, resulting in BKPyV-associated disease [34,88,89,90,91,92].

a. Transmission and persistence

Approximately 90% of BKPyV transmission is thought to occur during childhood through respiratory or oral transmission [93,94,95]. This hypothesis was supported by BKPyV seroconversion among 7 children at the time of an upper respiratory infection and detection of BKPyV DNA in respiratory tract or tonsillar tissues in other studies [94,95]. From the respiratory tract, the virus enters the bloodstream, infects peripheral blood leukocytes, and subsequently disseminates to various tissues [96]. Once acquired, BKPyV persists predominantly in urothelial cells although it has also been identified in lymphocytes and monocytes [97,98]. Although most BKPyV disease after allo-HSCT is thought to be a result of reactivation of previously acquired disease, there are reports of nosocomial BKPyV transmission [99,100,101]. By adulthood, more than 80% of the population is seropositive [102].

b. BKPyV-HC

**Figure 1:**
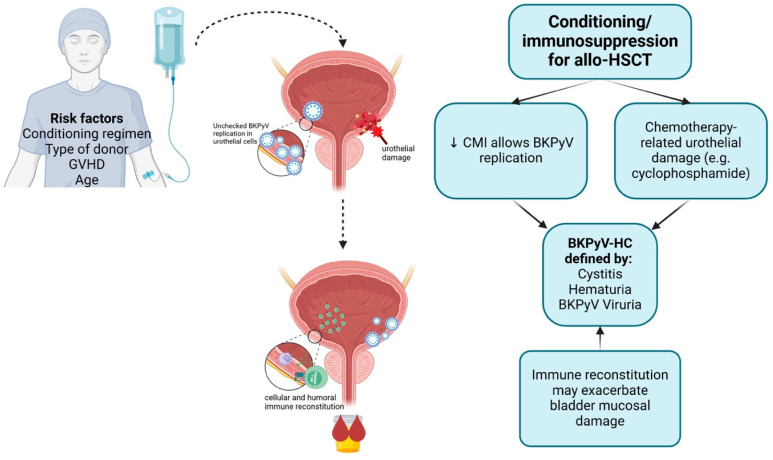
Three-stage model of BKPyV-HC pathophysiology. GVHD—graft versus host disease; allo-HSCT—allogeneic hematopoietic stem cell transplant; CMI—cell mediated immunity, BKPyV—BK polyomavirus; HC—hemorrhagic cystitis. Created in BioRender (Version December, 2024). Mendoza, M. (2025) https://BioRender.com/c75x266, accessed on 10 December 2024.

Determining the precise relationship between BKPyV and clinical disease or identifying the pathophysiology is hampered by the lack of histological data and lack of a precise definition of clinical disease. There are several theories of how BKPyV replication after HSCT results in BKPyV-HC. The first is that immunosuppression causes unchecked BKPyV replication, which has direct cytopathic effect on urothelial cells and mucosa [51,103] (Figure 1). Viral replication is particularly prominent in urothelial cells already damaged by prior therapies, such as cyclophosphamide. This replication, combined with the reduced activity of cytotoxic T cells, increases the risk of uncontrolled viral replication [55]. This theory is supported by studies demonstrating a relationship between viral load and HC severity [27,40,55] and studies demonstrating a relationship between lack of immune reconstitution and more severe or longer duration of BKPyV- HC [40,55].

Another theory suggests that immune reconstitution exacerbates mucosal damage by reacting to replicating virus or viral-induced changes to urothelial epithelium, potentiating further damage to bladder and urothelial tissues [26].

Leung et al. proposed a three-phase model for the development of BKPyV-associated HC. In the first phase, the conditioning regimen damages the bladder mucosa, creating a favorable environment for BKPYV replication. In the second phase, viral replication becomes unchecked due to impaired immunity. In the third phase, immune reconstitution and the return of anti-BKPyV immunity cause additional damage to the bladder mucosa [104] (Figure 1).

c. Future directions of study

Several questions remain regarding BKPyV-HC pathogenesis. It is unknown whether BKPyV replication detected in urine or blood represents replication in urothelial tissue, kidney tissue, or a combination of both [103], whether BKPyV causes nephropathy in the native kidneys of HSCT recipients even in the absence of HC [69,71,105], or how the type of immunosuppressive procedure (allo-HSCT vs KT) so strongly impacts the clinical manifestations of BKPyV replication (BKPyV-HC vs BKPyVAN).

In BKPyV-HC the relationship between BKPyV coming from renal epithelium vs bladder epithelium remains unclear [65,106] and, in contrast to BKPyVAN after KT, some studies have observed a nonlinear relationship between urine and plasma BKPyV load [40].

Similarly, the relationship between immune reconstitution and the occurrence and severity of BKPyV-HC has not been clearly established. Lastly, some definitions of BKPyV-HC include cystitis with microscopic hematuria and some definitions require Bedi grade ≥ 2 hematuria. It is unknown whether BKPyV DNAuria, cystitis symptoms, and microscopic hematuria represent a more mild form of BKPyV-HC or separate syndrome entirely, particularly as the degree of BKPyV DNAemia may be similar across groups of patients with cystitis symptoms regardless of the presence of macroscopic hematuria or clots [40,107].

### 2.3. Risk Factors for BKPyV-HC

Several factors associated with BKPyV-HC have been identified, which are often markers of higher severity of immunosuppression or lack of immune reconstitution.

These risk factors include: male sex, haploidentical, matched unrelated donors [43,52,108,109], myeloablative conditioning [43,52,110], acute/chronic GVHD [52], the presence of cytomegalovirus (CMV) reactivation or initiation of CMV treatment [52,109], CMV DNAuria [111], older age, use of lymphocyte depletion such as ATG, alemtuzumab, or T cell depleted grafts [45,112,113,114,115]. The subtype of BKPyV does not appear to be a risk factor for developing BKPyV-HC [116]. In the pediatric population, risk factors are similar to the adult patients including matched unrelated donors, a prior diagnosis of acute myeloblastic leukemia, and GVHD [117].

#### Post-Transplant Cyclophosphamide

Post-transplant cyclophosphamide (PTCy) has been increasingly adopted as GVHD prophylaxis following allo-HSCT [118] and has been identified as a risk for BKPyV-HC [119,120]. A study presented at the American Society of Hematology in 2024 reported an incidence of 72% at one hundred days post-HSCT, with a median onset of 30 days post-transplant. Notably, reducing the PTCy dose did not decrease the risk of BKPyV-HC but delayed its onset and reduced its duration [43]. Another retrospective study of patients receiving PTCy reported a median time to BKPyV-HC diagnosis of 29 days post-HSCT, with JCPyV coinfection detected in 24% of patients and cytomegalovirus DNAuria in 17%. Additionally, a higher prevalence of grade 3–4 HC was observed in younger patients and those who had a haploidentical donor [121].

The exact mechanisms by which PTCy elevates the risk are not fully understood. One hypothesis suggests that PTCy may cause a delay in immune reconstitution, affecting T cell recovery [122,123], leading to prolonged immunosuppression and creating an environment conducive to viral reactivation. Additionally, PTCy may induce direct urothelial toxicity, secondary to acrolein, a toxic metabolite of Cy [124], which could facilitate viral replication in the urinary tract [125].

### 2.4. Relationship of BKPyV DNAemia and HC with Immune System

Like other post-allo-HSCT viruses (e.g., Cytomegalovirus, Adenovirus), cellular immunity is thought to play a major role in the control of BKPyV. Viral variations in VP1 and LTag may escape control by neutralizing antibodies and T cells [16]. Sustained BK virus (BKPyV) DNAemia has been associated with impaired recovery of CD4+ and CD8+ T-cell subsets, possibly due to control by regulatory T cells [126,127,128,129]. Similarly, reconstitution of CD4+ and CD8+ cells is associated with BKPyV clearance in HSCT [128] and resolution of BKPyV-HC symptoms is associated with BKPyV-specific T cell responses [55]. At day 100, higher ALC, CD3, and CD8 counts were associated with lower hazard of BKPyV DNAuria in multivariable models [126]. Espada et al. characterized the recovery of BK virus–specific T-cell immunity in 77 adult patients with urinary symptoms after HSCT, comparing those with and without BKPyV DNAuria. Patients with BKPyV DNAuria had delayed CD4 T-cell recovery post-transplant but had faster recovery of BK virus–specific Th1 CD4 T cells, which were more frequent than cytolytic CD8 T cells. Among those with BKPyV DNAuria, patients with early reconstitution of BKPyV-specific interferon-γ+ and cytolytic CD4 T cells was linked to lower hematuria rates, highlighting a potential role in prevention of symptoms [130].

Humoral immunity likely also contributes to control of BKPyV replication; however, studies have not found a clear relationship between the presence of BKPyV-antibodies (which are not routinely obtained) and BKPyV-HC [131,132]. Studies in seropositive patients have demonstrated an association between high BKPyV IgG titers with subsequent BKPyV DNAuria, although this was not compared to seronegative patients [109]. Limited information exists regarding BKPyV syndromes in patients who were seronegative patients prior to transplantation. Among six children with BKPyV-HC after allo-HSCT, BKPyV viremia decreased as IgM levels initially increased, followed by IgG, suggesting the role of the humoral response in recovery [133,134].

### 2.5. Management of BKPyV

The two major approaches to BKPyV disease include symptom management and antiviral strategies intended to prevent or reduce BKPyV replication. There are no high-quality or randomized data to support any specific prophylaxis or management strategy for BKPyV-HC or other BKPyV-associated diseases. Among the therapies studied, intravenous (IV) and intravesical cidofovir were the most reported strategy, but no clear benefit was demonstrated, and the level of evidence has been graded low [42].

a. Supportive measures

Without significant data to support an antiviral strategy, supportive care is recommended for BKPyV-HC (grade AIII) [37]. Supportive measures include hydration, platelet transfusions, and analgesics [37]. HBO therapy has been explored in case series123 and case reports122,124,125. However, there is no good quality data to support its use.

b. Antiviral strategies

While the cornerstone of BKPyVAN management after kidney transplantation is reduction in immunosuppression (RIS), this is frequently not feasible after allo-HSCT due to the threat of donor alloreactivity and GVHD [37,67]. Several antivirals have been proposed or studied, including fluoroquinolones, leflunomide, cidofovir (IV or intravesical), brincidofovir, and virus-specific T cell (VST) therapy.

#### 2.5.1. Fluoroquinolones

Although quinolone antibiotics were initially thought to have modest anti-BKPyV activity in vitro and potential benefit in observational studies of BKPyV-HC after HSCT [135,136,137], three RCTs in KTRs have not shown antiviral effect either as prevention of BKPyV DNAuria or DNAemia. In one RCT of 3 months of levofloxacin vs placebo, BKPyV DNAuria was observed in 22/76 patients (29%) in the levofloxacin group and 26/78 patients (33.3%) in the comparison group [138]. In a second RCT, BKPyV viremia occurred in 25/133 patients (18.8%) in the ciprofloxacin group compared to 5/67 patients (7.5%) in the placebo group (*p* = 0.03) [139]. Lastly, among KTRs with BKPyV DNAemia the percentage of patients with BKPyV load reduction were 70.3% and 69.1% in the levofloxacin group (n = 22) and the placebo group (n = 21), respectively (*p* = 0.93) [140]. Several studies have also identified a higher risk of quinolone-resistant bacterial infections [135,138,139]. As a result, quinolones are not recommended for BKPyV treatment or prevention either after KT or HSCT [37,66,67].

#### 2.5.2. Leflunomide

Leflunomide is an antimetabolite drug with immunomodulatory and antiviral activity; its use has been supported by case reports and small retrospective studies [115,141,142]. However, a phase II study of a leflunomide derivative did not show substantial benefit in the treatment of BKPyVAN after KT as treatment in 30 patients led to a slightly greater reduction in urine BKPyV DNA load (3.1 log10 copies/μL in treatment group vs. 2.8 log10 copies/μL with placebo) and a small statistically significant reduction in viremia (1.9 log10 copies/μL in treatment group vs. 1.3 log10 copies/μL in placebo group, *p* = 0.049) [143]; leflunomide is not currently recommended [37,67].

#### 2.5.3. Cidofovir

Cidofovir is a nucleotide analog that inhibits a broad range of DNA viruses in vitro, although BKPyV does not possess its own polymerase. Its active metabolite has a long half-life of 15–65 h, allowing for administration at weekly intervals [37,144]. Intravenous (IV) or intravesical cidofovir have been commonly used for BKPyV-HC, but there is no consensus on the optimal dose, route, or frequency of administration [37]. When given IV, doses ranging from 3–5 mg/kg/week with probenecid, or lower doses of 0.5–1.5 mg/kg/week without probenecid are typically given [37]. To mitigate the risk of ocular and nephrotoxicity, cidofovir may be administered intravesically, although the potential for systemic exposure remains [145].

Small retrospective studies, case series, and case reports (without a comparator group) have suggested varying clinical response rates both intravenous (IV) and intravesical cidofovir in the treatment of BK polyomavirus-associated hemorrhagic cystitis (BKPyV-HC). Clinical response was generally defined as complete symptom resolution, while partial response (PR) referred to significant symptom improvement with persistent hematuria, though definitions varied across studies. Study sizes ranged from 4 to 57 patients in both pediatric and adult populations, with complete response (CR) rates between 60–85% for IV cidofovir and 59–92% for intravesical administration. A systematic review reported a CR in 117 of 172 patients (68%) treated with IV cidofovir and 15 of 17 patients (88.2%) treated with intravesical cidofovir [137,146,147,148,149,150,151,152,153,154,155,156,157,158,159,160].

However, there was inconsistency in the reporting and definitions of virologic responses, and other studies have demonstrated no reductions in BKPyV DNAemia associated with cidofovir administration [40,55,161]. Furthermore, a small randomized, placebo-controlled phase I/II trial of low-dose IV cidofovir given for treatment of BKPyVAN after KT did not significantly reduce BKPyV DNAemia [162].

#### 2.5.4. Brincidofovir

Brincidofovir (CMX001) is a lipid-conjugated prodrug of cidofovir with in vitro activity against a variety of double-stranded DNA viruses, including BKPyV. Importantly, brincidofovir is associated with lower rates of renal dysfunction compared to cidofovir [163]. Several case reports suggest potential benefits of this medication in managing BKPyV-associated conditions [164,165,166]. However, in a phase III study of brincidofovir for CMV prophylaxis after allo-HSCT, the percentage of patients with BKPyV infection was not different in the treatment vs placebo arms. Brincidofovir is not currently available [167].

#### 2.5.5. Virus-Specific T-Cells (VSTs)

VSTs are a targeted therapy used to treat viral infections in immunocompromised patients. Various approaches have been developed, including ex-vivo expansion of donor-derived VSTs or banking VSTs from healthy donors with diverse haplotypes for immediate infusion [168,169]. To address the multiple viral infections that affect immunocompromised patients, multi-virus VSTs have been developed and studied. Although adoptive transfer of VSTs is generally safe, the complexity of preparing these cells and their limited antiviral range have posed significant hurdles.

Case reports and series have reported favorable clinical outcomes including reduction in viral load and symptomatic improvement [170,171,172,173,174]. Two single arm, uncontrolled studies evaluated safety and efficacy of VSTs targeted against BKPyV. One study included 38 HSCT recipients and 3 SOT recipients with BKPyV DNAemia or BKPyV-HC; among patients with cystitis, 31/38 achieved complete response (CR); when assessing both BKPyV PCR levels and cystitis response, 22/38 patients achieved a CR; CR was defined as undetectable plasma BKPyV PCR within four weeks after the final VST infusion or resolution of hemorrhagic cystitis to below grade 2 without the need for medical therapy. No infusion-related toxicity, de novo GVHD, or organ rejection were found [175]. Another study of third-party BKPyV-specific cytotoxic T lymphocytes for patients with BKPyV-HC after allo HSCT demonstrated that by day 21, 33/56 patients achieved CR (defined as full resolution of symptoms and gross hematuria or a reduction in hemorrhagic cystitis (HC) grade from 2, 3, or 4 to 0 or 1), while by day 45, 34 of 49 patients achieved CR [176]. Post-infusion T cell detections have been observed and sustained for several months [170,173,176]. However, these promising results need to be confirmed in RCTs.

Posoleucel, an investigational multivirus-specific T-cell therapy, was found to be safe, well tolerated, and associated with greater reductions in BK viremia compared to placebo in a phase two study of 61 kidney transplant recipients [177]. However, a phase three study evaluating posoleucel for preventing viral infections after allo-HSCT, treating virus-associated hemorrhagic cystitis, and adenovirus were terminated early due to futility [178].

## 3. JCPyV and PML in HSCT Recipients

JCPyV is best known as a cause of PML due to different types of immunosuppression and has been reported as an uncommon complication after allo-HSCT [179]. JCPyV DNAuria and DNAemia are common after all-HSCT but less commonly associated with clinically significant disease. We review the clinical presentation, epidemiology, pathophysiology, and management below.

### 3.1. Clinical Presentation of JCPyV Syndromes After HSCT

a. Progressive multifocal leukoencephalopathy (PML)

Classically, the development of PML has been associated with specific types of immunosuppression, such as natalizumab or HIV/AIDS [180], however PML is seen among patients with immunosuppression of a variety of causes and occasionally among immunocompetent patients. Around 8% of cases of PML are made up of patients with hematological malignancies including HSCT recipients [181]. PML typically presents months to years after HSCT [181] and presenting symptoms are similar to those in other immunocompromised, non-allogeneic HSCT hosts: weakness, cerebellar symptoms, visual changes, aphasia, and hemiparesis [46,182,183,184,185]. The clinical diagnosis of PML relies on the presence of compatible symptoms, imaging features, and the presence of JCPyV by PCR from CSF; diagnosis may also be made based on histopathology with detection of JCPyV by electron microscopy, immunohistochemistry, or tissue PCR [186]. Detection of JCPyV in CSF alone without symptoms or radiographic signs of infection has poor predictive value for PML [187]. Cytological features of PML include hypertrophy of astrocytes, oligodendrocytes with enlarged, round nuclei, and disseminated foci of demyelination [180].

b. Viremia

Asymptomatic JCPyV DNAemia is uncommonly detected in blood samples of healthy adults [88], but is common after HSCT. Among 164 allo HSCT recipients who were retrospectively tested for JCPyV from whole blood samples originally sent for CMV, any DNAemia was detected in 40 (24%) and DNAemia in at least two samples was detected in 20 (12%); two patients were ultimately diagnosed with PML [188]. Other studies show a lower incidence (0–20%) of post-allo-HSCT viremia [181]. Among patients ultimately diagnosed with PML, JCPyV replication in peripheral blood preceded PML [183,188] but JCPyV detection in peripheral blood is an insensitive predictor as most patients with JCPyV viremia will not go on to develop PML.

c. JCPyV-associated renourinary syndromes

Asymptomatic JCPyV DNAuria is common both pre and post HSCT [181], and there is a positive quantitative relationship between DNA load in the urine and IgG index [181,189]. JCPyV is a recognized but uncommon cause of PyVAN after kidney transplant [18]. The true incidence of PyVAN after HSCT is unknown, and JCPyV DNAuria is less common after HSCT than BKPyV DNAuria [25], but it is possible that JCPyVAN could also affect HSCT recipients. BKPyV-HC is a well-described syndrome after HSCT; JCPyV-HC has been reported, but the incidence is unknown [190,191].

### 3.2. Pathogenesis of JCPyV Syndromes After HSCT

JCPyV is thought to spread ubiquitously throughout the population early in life through the respiratory or oral route; by the time of adulthood, 60–90% of the population is seropositive for JCPyV [90,192,193,194]. Following transmission and primary viremia, JCPyV persists primarily within the kidneys, and 20% of asymptomatic healthy individuals can intermittently shed JCPyV in their urine [180]. JCPyV can also be detected in tonsillar tissue, lymphocytes, and in brain tissue of healthy patients [194].

JCPyV is best known as a cause of PML but has also been reported as a cause of encephalopathy, meningitis, granule cell neuronopathy, PyV associated nephropathy (PyVAN), or HC [18,67,194]. PML is thought to arise when immunosuppression allows for JCPyV replication within the kidney, hematogenous spread via infected lymphocytes to the central nervous system, and replication within glial cells in the CNS [180,194].

The pathophysiology of PML after allo-HSCT is thought to be similar to PML associated with other forms of immunosuppression. Genotypic differences in the NCCR between JCPyV associated with PML (“prototypical” JCPyV) and JCPyV detected in urine and sewage (“archetypal” JCPyV) have been observed, giving rise to multiple hypotheses regarding pathogenesis. Archetypal JCPyV is thought to be the strain transmitted from person to person, which then persists in tonsils, renal cells, and other tissues. It is unknown whether genetic rearrangements in the NCCR occur in peripheral tissues, in the CNS, or in both to produce the prototypical strain of JCPyV. However, immunosuppression is thought to permit prototypical JCPyV to spread to or replication within the CNS, potentially via generation of VP1 escape mutant strains that confer resistance to circulating VP1 antibodies [180,194,195,196,197]. The degree of mutations (number of repeats) within the NCCR has been correlated with poorer PML prognosis [195,198,199].

The control of JCPyV replication and PML has been strongly associated with cellular immunity, although humoral immunity likely also plays a role [13,200]. In a small cohort of HSCT recipients prospectively monitored pre- and post-transplant, the presence of JCPyV DNAemia inversely correlated with detection of cellular immune responses; JCPyV-specific CD4+ and CD8+ T cell responses increased 12–18 months post-HSCT [181]. In another small case series of patients with PML (none post-HSCT), however, plasma neutralizing antibody levels were highest among PML survivors [197].

### 3.3. Management of PML

As below with BKPyV-associated diseases, there is no demonstrated effective treatment for JCPyV disease, including PML, and the cornerstone of management is immune restoration [197]. Numerous medications have been investigated for their antiviral activity including via large-scale drug screens [201], some with mechanisms that could disrupt known components of the JCPyV life cycle, including trifluoperazine (calcium signaling-related inhibition), topotecan (topoisomerase inhibitor), cytarabine (polymerase inhibition), mefloquine (antagonize endosomal acidification), mirtazapine (interrupt viral attachment/entry and trafficking), pembrolizumab or nivolumab (boost cellular immune activity), and cidofovir/brincidofovir (polymerase inhibition) [201,202]; however many medications have had mixed outcomes or lack of benefit in larger observational studies [17,180,203,204,205,206,207,208,209].

JCPyV- or BKPyV-specific T cells (leveraging cross reactivity due to virus similarity) have been used successfully in patients with PML, including after HSCT, and a few studies have examined the impact of VSTs in multiple patients with PML [210,211,212]. In the first, nine patients with definite PML received JCPyV-specific T cells (autologous or allogeneic HLA-matched); 6/9 patients achieved control of their JCPyV, evidenced by symptomatic stability or improvement and a lower JC load in CSF [213]. In a second study of 4 HSCT (2 autologous, 2 allogeneic) recipients with PML, no cases of graft versus host disease or infusion reactions were associated with transfer of third party BKPyV-directed VSTs. One patient’s disease clinically stabilized, with clearance of JCPyV from blood and CSF, but the other three patients had progressive neurologic decline despite some improvement JCPyV load [214]. Lastly, 28 patients received BKPyV-directed VSTs isolated from healthy donors; 22/28 (79%) demonstrated clinical stabilization/improvement and a reduced JCPyV load over >1 year of follow up. Three of 4 enrolled allo-HSCT recipients responded. Survival was significantly higher (hazard ratio, 0.42, *p* = 0.02) in comparison to a historical control group (n = 113) [215]. These results are promising and should be further examined in larger, well-controlled studies.

## 4. Other Human Polyomavirus (HPyV) Syndromes After Allo-HSCT

Fifteen PyVs have been isolated from humans—around half have been linked with clinical disease; Karolinska Institute PyV (KIPyV), Washington University PyV (WUPyV), Merkel cell PyV (MCPyV), HPyV6, HPyV7, Trichodysplasia Spinulosa PyV (TSPyV) [17]. A similar disease paradigm to JCPyV and BKPyV exists for the remainder of the common HPyVs: viral acquisition is thought to occur early in life, detection of HPyV can occur in healthy, asymptomatic individuals, and immunosuppression is thought to permit increased replication and the potential for symptomatic disease. TSPyV may be the exception as some data suggest that primary infection is associated with symptoms in at least some cases [216].

Several HPyVs are hypothesized to be a cause of clinical syndromes in immunosuppressed individuals due to reports of detection, but causality has yet to be definitively established: HPyV 6 and HPyV 7 (identified in cases of pruritic dermatoses) [217,218] and KIPyV and WUPyV (identified in cases of respiratory infection). Other HPyVs have been causally linked with clinical manifestations; these include MCPyV (cause of Merkel cell carcinoma; reported after HSCT) [8] and TSPyV (trichodysplasia spinulosa, characterized by spiny follicular papules and projections; cases reported after organ transplant but not allo-HSCT) [219].

Subclinical detection from patients undergoing allo-HSCT is common from plasma or other samples. In a study that performed regular plasma sampling for 109 consecutive allo-HSCT recipients, several HPyVs were detected at least once at or after HSCT, including HPyV6 (24% of patients), HPyV7 (14% of patients), and MCPyV (21%); HPyV9 and TSPyV were not detected in any samples [34]. A similar study in pediatric patients also had a low rate of detection of TSPyV (1.9%) [220]. HPyV7 has been identified in urine samples of one patient following allo-HSCT along with BKPyV [221].

Although KIPyV and WUPyV are not commonly detected in plasma sampling [222], they have each been detected in 5–20% of allo-HSCT recipients with regular upper respiratory sampling [223,224,225]. Detection of WU or KI (relative to no detection) was associated with some respiratory symptoms (e.g., wheezing, sputum production) [225]. WUPyV is suspected to have caused respiratory disease and death in one reported patient after allo-HSCT [226]. The diagnosis was based on molecular detection of WUPyV, WU-VP1-specific immunohistochemical detection, and electron microscopy visualization of viral capsids of compatible size within lung tissue from autopsy specimens.

There are insufficient data to guide treatment in cases where clinical disease is suspected, though cases of treatment with valganciclovir, cidofovir, and reduction in immunosuppression are reported [227].

## 5. Conclusions and Future Directions

Polyomavirus-associated diseases, particularly BKPyV-HC and JCPyV-PML, remain significant complications in allo-HSCT recipients, with limited effective therapies and high morbidity. Future research should focus on establishing the precise pathophysiology of PyV reactivation, including the role of immune reconstitution and host-virus interactions. Development of targeted antiviral agents and virus-specific T-cell therapies shows promise but requires validation in controlled trials. Improved diagnostic strategies, including standardized thresholds for DNAemia and DNAuria, are needed to enhance early detection and risk stratification. Finally, understanding the interplay between immunosuppression, viral pathogenesis, and host immunity may pave the way for innovative prophylactic and therapeutic approaches.

## Data Availability

No new data were created or analyzed in this study. Data sharing is not applicable to this article.
